# Digital health intervention in patients undergoing cardiac rehabilitation: systematic review and meta-analysis

**DOI:** 10.12688/f1000research.152315.1

**Published:** 2024-06-07

**Authors:** Ali Suleiman Harbi, Kim Lam Soh, Putri Binti Yubbu, Kim Geok Soh

**Affiliations:** 1 Department of Nursing, Faculty of Medicine & Health Sciences,, University Putra Malaysia, Serdang, Selangor, 43400, Malaysia; 2 Department of Paediatrics, Faculty of Medicine & Health Sciences,, University Putra Malaysia, Serdang, Selangor, 43400, Malaysia; 3 Department of Sport Studies, Faculty of Educational Studies, University Putra Malaysia, Serdang, Selangor, 43400, Malaysia

**Keywords:** Digital, virtual, telerehabilitation, mhealth, wearable devices, cardiac rehabilitation

## Abstract

**Background:**

Cardiovascular disease (CVD) continues to be the foremost mortality internationally. Cardiac rehabilitation has proven as an effective program in reducing CVD burden. Participation in cardiac rehabilitation programs is very low. Digital health intervention emerged as an alternative method to deliver Cardiac rehabilitation. This review aimed to investigate the impact of digital health intervention on the outcomes of interest

**Methods:**

the following databases: PubMed, CINAHL, Scopus, and Cochrane Library have been searched to retrieve randomized controlled trials that examine the impact of digital health intervention on blood pressure, body mass index, lipid profile, blood glucose, Six-Minute Walk Test, and peak oxygen consumption. filters were set to include studies published in English between 2000-2023.

**Results:**

Nineteen studies were included in this review. Six-Minute Walk Test (MD = 16.70; 95% CI: 6.00 to 27.39, p = 0.000) and maximal oxygen consumption (SMD = 0.27; 95% CI: 0.08 to 0.45, p = 0.004) significantly improved following digital health intervention, after employing the sensitivity analysis significant improvement was observed in systolic (MD = -2.54; 95% CI: -4.98 to -0.11, p = 0.04) and diastolic blood pressure (SMD = -2.0182; 95% CI: -3.9436 to -0.0928, p = 0.04) favoring experimental groups. Subgroup analysis revealed significant improvement in quality of life after three months of follow-up (SMD = 0.18; 95% CI: 0.05 to 0.31, p = 0.00), no significant differences have been observed in body mass index, lipid profile, and blood glucose.

**Conclusion:**

The findings emphasize the significant impact of digital vs CBCR or usual care on physical capacity, blood pressure, and quality of life. Despite the non-statistically significant differences in body mass index and lipid profile, the comparable effect between the two methods suggests the superiority of digital over CBCR or usual care due to its convenient nature, accessibility, and cost-effectiveness.

## Introduction

Cardiovascular disease (CVD) continues to be the foremost cause of morbidity and mortality internationally, ^1^^,^^2^ contributing to a substantial health challenge and economic burden in all global regions. ^3^ In 2020, nearly 19 million deaths were linked to CVD, representing an 18.7% increase from 2010, and over half a billion individuals around the world are struggling with CVDs. ^4^ Despite the evolution of various technologies used in the treatment of CVD over recent decades, patients still commonly experience subsequent cardiovascular events, such as stroke and myocardial infarction, leading to recurrent hospital admissions, and raising the personal, social, and economic burden of the disease. ^5^ Therefore, presence of continuous management after diagnosed with CVD is very important.

Cardiac rehabilitation (CR) has proven as an effective program in reducing CVD burden. ^6^ A growing body of evidence highlights the positive impact of CR in controlling the modifiable risk factors, such as obesity, smoking, sedentary lifestyle, hyperglycemia, and hypertension. ^7^ Unfortunately, participation in CR programs (CRPs) is very low globally, less than 20% of eligible patients refer to CRPs. ^8^^,^^9^ Common barriers that prevent enrollment in and adherence to CRPs include a lack of transportation, the nature of the disease, and personal and job responsibilities. ^10^^,^^11^ Moreover, such programs were profoundly affected during COVID-19 lockdowns, entailing escalating healthcare costs due to reduced healthcare service provision, including detection of CVD problems, long-term management, and increased risk due to sedentary behaviors during the period 2020-2022. ^12^

In order to overcome these existential barriers, healthcare systems have started thinking about alternative methods to deliver CRPs, including using digital health intervention (DHI) as a medium to deliver CRPs. ^13^^–^^16^ Different types of DHI have been used, such as, smartphone applications, internet websites and platforms, virtual reality, short messages, and wearable and sensor devices. ^16^^,^^17^ Innovative ideas were applied using these technologies to monitor, coach, and track patients remotely during CRPs. The spread of smartphones and the availability of low-price internet in many contexts enables healthcare providers to design several types of CRPs. ^13^

Recently, the World Heart Federation Roadmap for Digital Health in Cardiology emphasized the capacity of digital health technologies to realize optimal and universal health coverage. It seeks to encourage both patients and healthcare providers, endorsing universal health coverage, improving long-term patient outcomes and care experiences, and mitigating healthcare costs. ^18^

The nonstop advancement in technology has introduced novel DHI interventions, that warrant a comprehensive assessment of their effect on the outcomes for patients undergoing CR. the presence of new studies in this field, providing an opportunity to consolidate and analyze the expanding body of literature. Moreover, the presence of varying results in previous works emphasizes the necessity for a rigorous meta-analysis to clarify consistent patterns, recognize potential sources of heterogeneity, and offer valuable perspectives into refining DHI strategies for CR. By synthesizing these elements, this review aims to contribute a deep understanding of the role of digital health intervention (DHI) in enhancing cardiometabolic risk factors, physical capacity, and quality of life (QoL), guiding both researchers and healthcare providers toward evidence-based interventions and optimizing patient outcomes in the context of CR.

## Methods

### Search strategy

This systematic review and meta-analysis followed the Preferred Reporting Items for Systematic Reviews and Meta-Analysis (PRISMA) guidelines. ^19^ Two authors (ASH and PY) independently searched the following databases: PubMed, CINAHL, Scopus, and Cochrane Library. References of included studies also were searched manually for studies not included in primary search. The time filter was set to retrieve studies between 2000-2023, and the language filter was set to include only studies published in English. The two authors used Medical Subject Headings (MeSH) to determine the relevant alternative keywords used in the search process. The retrieved papers were initially checked by titles for potential inclusion in this systematic review and meta-analysis, and those which passed this stage were sequentially checked for eligibility by reading their abstracts and then full texts. Any conflict was resolved by discussion until consensus was reached among the authors, and the final decision about the included and excluded studies was made by the senior author (SKL).

### Inclusion and exclusion criteria

The inclusion criteria of this systematic review and meta-analysis entailed that included studies: (1) were randomized controlled trials; (2) studied populations of patients with CVD; (3) studied populations aged ≥ 18 years; (4) employed interventions using digital technology; (5) investigated the impact of DHI on one or more of the outcomes of interest; and (6) had follow-up periods of at least four weeks. Any study did not meet these criteria was excluded. In addition, studies with mixed populations (i.e., patients with CVD and patients with non-CVD) were excluded. Duplicate retrieved studies were removed using. The EndNote software (Endnote X9), and then by manual checking.

### Data extraction and analysis

Data were extracted and recorded in a preset Microsoft Excel spreadsheet independently by one reviewer (ASH), and were then checked by another reviewer (PY). The extracted data included: first author’s name; year of publication; sample size and number of participants in each group; type of population; type of DHI; outcomes included in the review; change in mean, standard deviation, for experimental and control groups for each outcome; country where the study was conducted; mean participants’ age; and percentage of each gender. Units were converted when necessary. For example, if a study presented cholesterol in mg/dl, this was converted into mmol/L.

The intended population for this review and meta-analysis comprised patients with CVD, including those who were diagnosed with heart failure, myocardial infarction, and those who had undergone percutaneous intervention, coronary artery bypass graft, or valvular surgery. The considered DHIs included special applications delivered via smartphone, social media, and the web, using wearable devices and sensors, and using virtual technology. The comparator is the control group who revived center-based CR (CBCR) or usual care. The outcomes of interest for this study are cardiometabolic risk factors, physical capacity and QoL, specifically: systolic blood pressure (SBP), diastolic blood pressure (DBP), body mass index (BMI), total cholesterol (TC), low density lipoprotein (LDL), high density lipoprotein (HDL), triglyceride (TG), blood glucose (BG), Six-Minute Walk Test (6-MWT), and peak oxygen consumption (VO _2 peak_).

Data analysis was performed in accordance with Cochrane handbook for systematic reviews of interventions. ^20^ Using Jamovi software (Jamovi 24.11). Random effect statistical model was applied. To estimate the effect of DHI versus CBCR or usual care, change in means from baseline (change score) and standard deviations were used. Heterogeneity was tested using I ^2^, whereby values of 25%, 50%, and 75% indicate low, moderate, and high heterogeneity, respectively. Hypothesis testing was performed at a two-tailed 0.05 level and a 95% CI.

If I ^2^ values showed high heterogeneity (I ^2^ > 50%), sensitivity analysis was performed by removing the study(s) that caused heterogeneity. Publication bias was assessed visually by a funnel plot and statistically by the Egger test. Subgroup analysis was performed based on duration of follow-up (≥ 3 months vs ˂ 3 months), population average age (≥ 60 vs ˂ 60 years) and medium of delivery (smartphone vs other methods).

### Risk of bias

the Cochrane risk-of-bias tool for randomized trials (RoB 2) ^21^ was used to assay risk of bias. Two authors (ASH and PY) independently evaluated the included studies based on the following domains: (1) random sequence generation (selection bias); (2) allocation concealment (selection bias); (3) blinding of outcome assessment (detection bias); (4) incomplete outcome data (attrition bias); and (5) selective reporting (reporting bias). RoB 2 provides signal questions for each domain, which the evaluator should use to make an overall Risk of bias judgement (which can be low, high, or unclear). Blinding of participants and personnel is impossible due to the intrinsic nature of rehabilitation studies, so this domain was not assessed.

### Certainty of evidence assessment

The certainty of evidences was assessed using Grading of Recommendation Assessment, Development and Evaluation (GRADE) approach, GRADE is a systematic approach to rating the certainty of evidence in systematic reviews, the users rate the quality of evidence for each outcome of interest based on five criteria (i.e. risk of bias, inconsistency, indirectness, imprecision, and publication bias) in all studies that reported that particular outcome. according to which the quality of evidence could be high, moderate, low, or very low. ^22^ RCTs are considered by the tool to be generally high quality evidence, but they may be downgraded due to the assessment of Risk of bias, inconsistency, indirectness, imprecision, and publication bias.

## Results

### Search results

**Figure 1.**
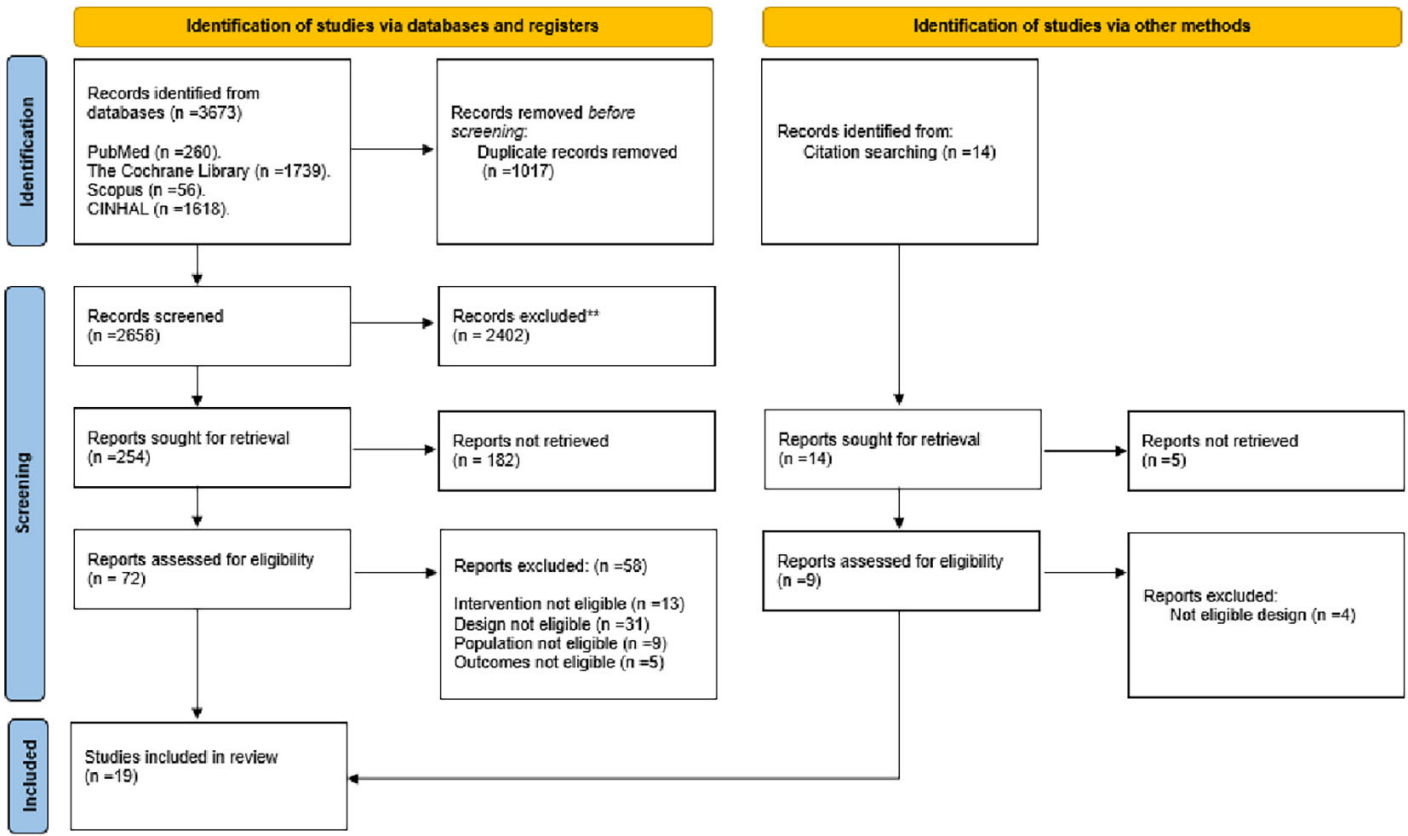
PRISMA flow diagram of literature search process.

The selected databases (PubMed, Cochrane Library, CINHAL, and Scopus) were last searched on 3 ^rd^ October 2023, and the bibliographies of included studies were also reviewed to identify other related works. The searching process revealed 3673 studies, 2656 of which remained after removing duplicates. Subsequently, 254 remained after reviewing titles, and 173 after reviewing abstracts. Consequently, 81 were eligible for full-text reviewing, and the final number of studies included in this systematic review and meta-analysis was 19. Figure 1 illustrates the searching process.

### Characteristics of included studies

This systematic review and meta-analysis of 19 RCTs published between 2014 and 2023 encompassed 1740 participants. The included studies were conducted in 11 countries (Australia, China, Czech Republic, Finland, Japan, the Netherlands, New Zealand, Poland, Portugal, Spain, and the USA). Sample sizes ranged from 22 to 312, and duration of follow-up ranged from 6 weeks to 12 months. The outcomes reported in the studies were SBP (n = 12), DBP (n = 10), BMI (n = 10), TC (n = 9), LDL (n = 9), HDL (n = 7), TC (n = 6), Qol (n = 10), BG (n = 3), VO _2 peak_ (n = 7), and 6-MWT (n = 5). Participants were diagnosed with CHD, myocardial infarction, having undergone percutaneous intervention, valve surgery, or coronary artery bypass graft. Table 1 demonstrates the studies’ characteristics.

**Table 1.** Characteristics of included studies.

Author Year Country	Sample size Female (%)	Population Diagnosis	Type of Technology	Duration	Outcomes	P value
(Ögmundsdóttir Michelsen et al., 2022) Sweden	144 (24 %)	patients who had MI	web-based application	14 months	SBP	0.22
					DBP	0.69
					BMI	0.57
					TC	0.41
					LDL	0.20
(Maddison et al., 2019) New Zealand	162 (14 %)	Patients with CHD	web-based platform and	24 weeks	SBP	NS
					DBP	NS
					BMI	NS
					TC	NS
					LDL	NS
					HDL	NS
					TG	NS
					VO _2 peak_	NS
					QoL	NS
					BG	NS
(Fang et al., 2019) China	67 (37 %)	Patients After PCI	smartphone application, wearable sensor and computer servers,	6 weeks	SBP	0.139
					DBP	0.552
					6MWT	0.006
(Widmer et al., 2017) USA	71 (18 %)	Patients after PCI	Smartphone app and web-based portal	3 months	SBP	0.67
					DBP	0.93
					BMI	0.01
					TC	0.24
					LDL	0.26
					HDL	0.73
					TG	0.36
					BG	0.88
					VO _2 peak_	0.67
(Claes et al., 2020) Belgium and Ireland	120 (18.3 %)	Patients with CVD	A web-based system	6 months	SBP	0.10
					DBP	004
					BMI	0.23
					TC	0.15
					LDL	0.12
					HDL	0.29
					TG	0.31
					BG	0.48
					VO _2 peak_	.64
(Leandro et al., 2021) Brazil	39 (73 %)	patients with hypertension.	Virtual cardiovascular rehabilitation (VCR) program	12 weeks	SBP	0. .554
					DBP	0. .131
					BMI	0. .401
					6MWT	0. .001
(Su & Yu, 2021) China,	146 (83.6 %)	Patients with CHD	E-platform pedometer-based monitoring Social media (WeChat)	12 months	SBP	0. .944
					DBP	0. .599
					BMI	0. .181
					QoL	0. .593
(Varnfield et al., 2014) Australia	120 (13 %)	Patients with CVD	Smartphone platform	6 weeks	SBP	0. .2
					DBP	0.05
					TC	0. .07
					LDL	0. .3
					HDL	0. .4
					TG	<0.03
					6MWT	<0.001
					QoL	<0.001
(Johnston et al., 2016) Sweden	174 (19 %)	Patients after MI	Smartphone platform	6 months	SBP	.749
					BMI	.366
					LDL	.004
					QoL	.059
(Lunde et al., 2020) Norway	113 (22.1 %)	Patients with CVD	Smartphone platform	12 months	SBP	NS
					DBP	NS
					TC	NS
					LDL	NS
					HDL	NS
					TG	NS
					VO2peak	<0.05
(Dorje et al., 2019) China	312 (19 %)	Patients after PCI	Social media WeChat and built-in pedometer	12 months	SBP	0·029
					BMI	0·14
					TC	0·018
					LDL	0·016
					HDL	0·27
					TG	0·26
					6MWT	0·027
					QoL	0·51
(Skobel et al., 2017) German, British and Spanish,	61 (11 %)	Patients with CAD	The Professional System) a Web-based tool) The Patient Station (gateway) The Portable Station (sensor and a smartphone)	6 months	SBP	0.003
					DBP	0.01
					BMI	0.56
					TC	0.64
					LDL	0.57
					HDL	0.55
					BG	0.11
					QoL	0.98
(Barnason et al., 2019) USA	43 (30 %)	Patients after PCI or CABS.	Telephone coaching	12 weeks	BMI	NS
(Vieira et al., 2018) Portugal	22 (0 %)	Patients with CAD	Virtual reality	6 months	QoL	NS
(Batalik et al., 2020) Czech Republic	51 (18 %)	Patients with CVD	wrist monitor	12 months	VO2peak	59
					QoL	.56
(Nagatomi et al., 2022) Japan	30 (7 %)	Patients with CHF	web application wearable device	3 months	6MWT	< 0.001
					QoL	0.26
					TC	0.02
(Duscha et al., 2018) USA	25 (24 %)	Patients with CVD	Smartphone application	12 weeks	VO2peak	NS
(Lahtio et al., 2023) Finland	59 (19 %)	Patients with CVD	software application	12 months	BMI	NS
					6MWT	NS
(Song et al., 2020) China	96 (14 %)	Patients with CHD	smartphone software and heart rate belts	6 months	VO2peak	0.034

6-MWT = Six-Minute Walk Test, BMI = body mass index, DBP = diastolic blood pressure, HDL = High-density lipoprotein, LDL = low-density lipoprotein, QoL = quality of life, SBP = systolic blood pressure, TC = total cholesterol, TG = triglyceride, VO _2 peak_ = peak oxygen consumption.

### Interventions

Studies included in this review reported different types of CR. Five studies employed only exercise CR, ^23^^–^^27^ while others employed comprehensive multicomponent forms. Six studies applied DHI plus CBCR. ^25^^,^^28^^–^^32^

Different devices were used to deliver DHI in the included studies, of which the smartphone was the most widely used, through special applications or platforms equipped with instructional features. ^28^^,^^30^^,^^33^^–^^37^ Patients could use these programs independently, while healthcare professionals could also use and monitor them to determine participants’ status and performance. They could also interact, provide coaching, and give instant or scheduled feedback to patients, and use text messages, emails, and common social media messaging formats to interact with participants.

Smartphones were also used in combination with wearable devices in many studies, such as step trackers, wrist heart rate monitors, biometric vests, and wearable belts. ^24^^–^^27^^,^^31^^,^^38^^–^^42^ The wearable devices had sensors providing information about heart and respiratory rates, ECG, time, training mode, duration, and distance of training physical activity. Some of the devices were connected to participants’ smartphones, computers, or landlines, ^29^ enabling researchers to monitor participants. Educational information about patients’ healthy lifestyles and dietary habits was also provided through technology used in some studies, in which participants were asked to enter their values for metrics such as BP, lipids, glucose, and weight. ^30^^,^^31^

Two studies, ^23^^,^^43^ used virtual reality technology to provide DHI. Virtual reality is a unique technology fitted to fulfill many requirements for effective rehabilitation. ^44^ It can create a fully virtual and three-dimensional setting, where the user interacts through different sensory stimuluses, forming as much of the reality as possible, for the purpose of encouraging participants to increase their physical activity levels, using various materials like videos and games. ^45^

### Risk of bias in included studies

All 19 included studies were assessed for Risk of bias; none of them had high Risk of bias for the random sequence generation domain; six (30%) had unclear Risk of bias, since they did not report the method of randomization; and the remaining 13 studies (70%) were assessed as having low Risk of bias. Five studies did not report sufficient information about allocation concealment and were assessed as having unclear bias, while the remaining 14 mentioned that they used concealed opaque envelopes to allocate participants. For the blinding outcome assessment domain, 10 (50%) studies did not provide sufficient information on whether the data was analyzed blindly or not so, they were assessed as unclear Risk of bias, while the remaining 9 studies mentioned that the data was analyzed by a blinded statistician.

**Figure 2.**
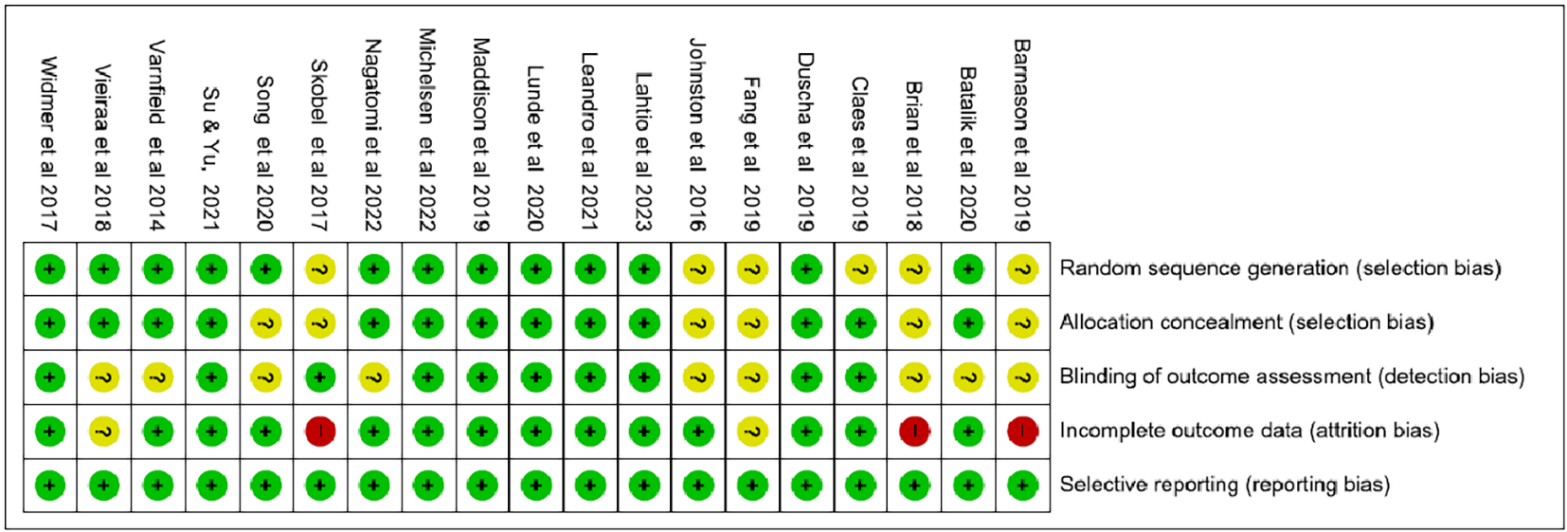
Risk of bias summary.

**Figure 3.**
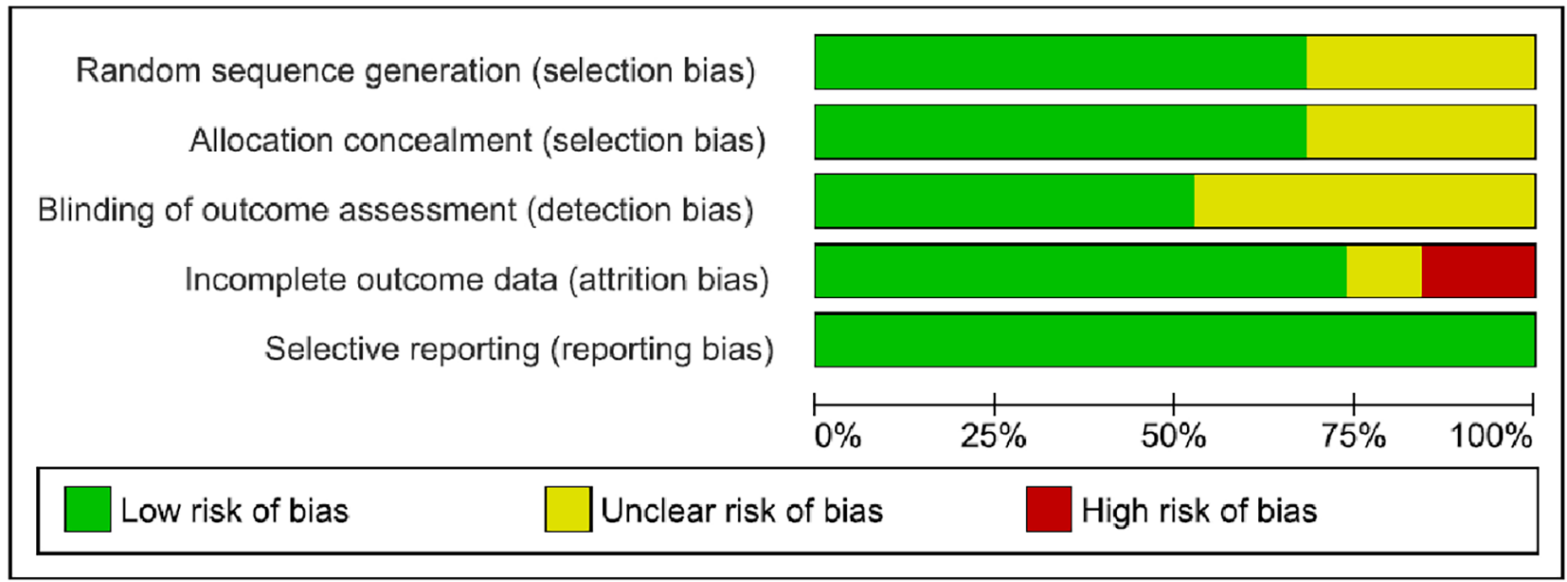
Risk of bias graph.

For incomplete outcome data, 3 (15%) studies were assessed as high Risk of bias because of the high dropout rate during the intervention, and they did not report how they treated the missing data; and 2 (10%) studies were assessed as unclear Risk of bias, with attrition rates >10%. The remaining 14 (75%) reported the use of intention-to-treat (ITT) analysis and detailed the methods to treat the missing data. All studies were assessed as low Risk of bias regarding selective reporting bias. ( Figure 2) and ( Figure 3) summarize the outcomes of bias testing.

### Certainty of evidence

The certainty of evidence was assessed using the GRADE approach. Downgrading was undertaken in some cases, mainly due to imprecision, because some included studies had small sample sizes. Of the eleven outcomes examined in this review, nine outcomes were judged as having moderate quality of evidence (SBP, DBP, TC, LDL, HDL, TG, VO _2 peak_, 6-MWT, and QoL); one outcome (BMI) was judged as a low quality of evidence, and another (BG) was judged as very low quality of evidence. Details of assessing the certainty of evidence are presented in Table 2.

**Table 2.** Certainty of evidence.

Outcome	No. studies		Risk of bias	Inconsistency	Indirectness	Imprecision	Publication bias	Certainty
	No. participants							
	(ex n =)	(co n =)						
SBP	12		Not serious	Not serious	Not serious	Downgrade	Downgrade	⨁⨁⨁◯ Moderate
	(730)	(679)						
DBP	10		Not serious	Not serious	Not serious	Downgrade	No bias	⨁⨁⨁◯ Moderate
	494	451						
BMI	10		Downgrade	Not serious	Not serious	Downgrade	No bias	⨁⨁◯◯ Low
	531	539						
TC	10		Not serious	Not serious	Not serious	Downgrade	No bias	⨁⨁⨁◯ Moderate
	501	457						
LDL	9		Not serious	Not serious	Not serious	Downgrade	No bias	⨁⨁⨁◯ Moderate
	520	470						
HDL	7		Not serious	Not serious	Not serious	Downgrade	No bias	⨁⨁⨁◯ Moderate
	384	398						
TG	6		Not serious	Not serious	Not serious	Downgrade	No bias	⨁⨁⨁◯ Moderate
	365	358						
6-mwt	5		Not serious	Not serious	Not serious	Downgrade	No bias	⨁⨁⨁◯ Moderate
	257	239						
VO _2 peak_	7		Not serious	Not serious	Not serious	Downgrade	No bias	⨁⨁⨁◯ Moderate
	284	300						
QoL	10		Not serious	Not serious	Not serious	Downgrade	No bias	⨁⨁⨁◯ Moderate
	533	544						
Blood glucose	3		Not serious	Downgrade	Not serious	Downgrade	Downgrade	⨁◯◯◯ Very low
	163	153						

6-MWT = Six-Minute Walk Test, BMI = body mass index, DBP = diastolic blood pressure, HDL = High-density lipoprotein, LDL = low-density lipoprotein, QoL = quality of life, SBP = systolic blood pressure, TC = total cholesterol, TG = triglyceride, VO _2 peak_ = peak oxygen consumption.

### Meta-analysis of included studies

The pooled effect siize was calculated using mean difference in case that all studies used the same scale and using hedge’s g in case different scales were used based on the following formulas

g^=∑i=1KWigi∑i=1KWi

Where g^ is the pooled effect size, K is the number of studies, g _i_ is the effect size of study i, and W _i_ is the weight for study i

$$\mathrm{MD}=\frac{\sum_{i=1}^{K}\mathrm{MDi}}{\sum_{i=1}^{K}\mathrm{Wi}}$$

Where MD is the pooled mean difference, K is the number of studies, MD _i_ is the weight for study i, and W _i_ is the effect size of study i

*Blood pressure*

**Figure 4.**
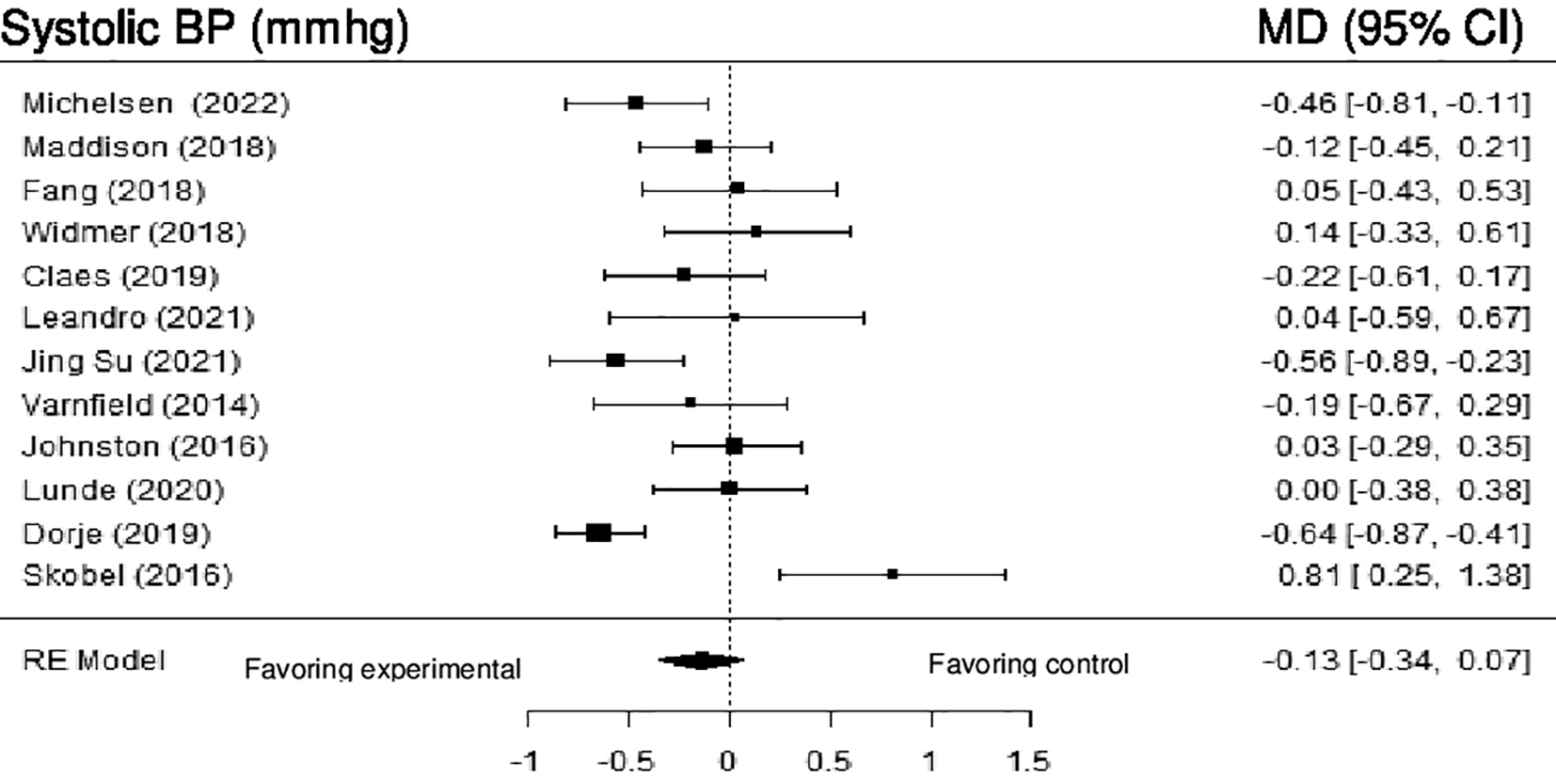
Forest plot of DHI vs CBCR/usual care impacts on SBP.

**Systolic blood pressure:** 12 studies evaluated the effects of DHI on SBP, and meta-analysis showed no statistically significant difference (MD = -2.16; 95% CI: -5.41 to 1.08), with high heterogeneity (Q (11) = 40.05, p < 0.0001, tau ^2^ = 0.09, I ^2^ = 71.53%). After employing the sensitivity analysis, by removing the studies by Skobel et al. (2017) and Dorje et al. (2019), the result became statistically significant (MD = -2.54; 95% CI: -4.98 to -0.11) (z = -2.05, p = 0.04), with moderate heterogeneity (Q (9) = 12.67, p = 0.18, tau ^2^ = 5.18, I ^2^ = 34.69%). Figure 4 shows the forest plot of the impact of DHI vs CBCR/usual care on SBP.

**Figure 5.**
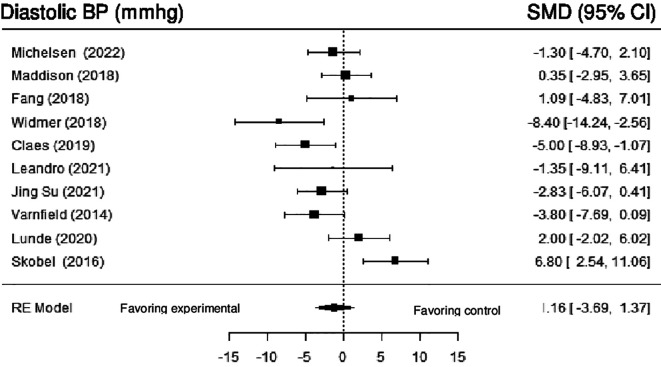
Forest plot of DHI vs CBCR/usual care impacts on DBP.

**Diastolic blood pressure:** 10 studies evaluated the effects of DHI on DBP. Meta-analysis showed no statistically significant difference (SMD = -1.16; 95% CI: -3.69 to 1.37) (z = -0.90, p = 0.3681), with high heterogeneity (Q (9) = 29.51, p = 0.0005, tau ^2^ = 11.45, I ^2^ = 71.96%). After employing the sensitivity analysis, by removing the study by Skobel et al. (2017), the result became statistically significant, with moderate heterogeneity (SMD = -2.0182; 95% CI: -3.9436 to -0.0928) (z = -2.05, p = 0.04) (Q (8) = 14.91, p = 0.0608, tau ^2^ = 3.83, I ^2^ = 46.29%). Figure 5 shows the forest plot of the impact of DHI vs CBCR/usual care on DBP.

*Lipid profile*

**Figure 6.**
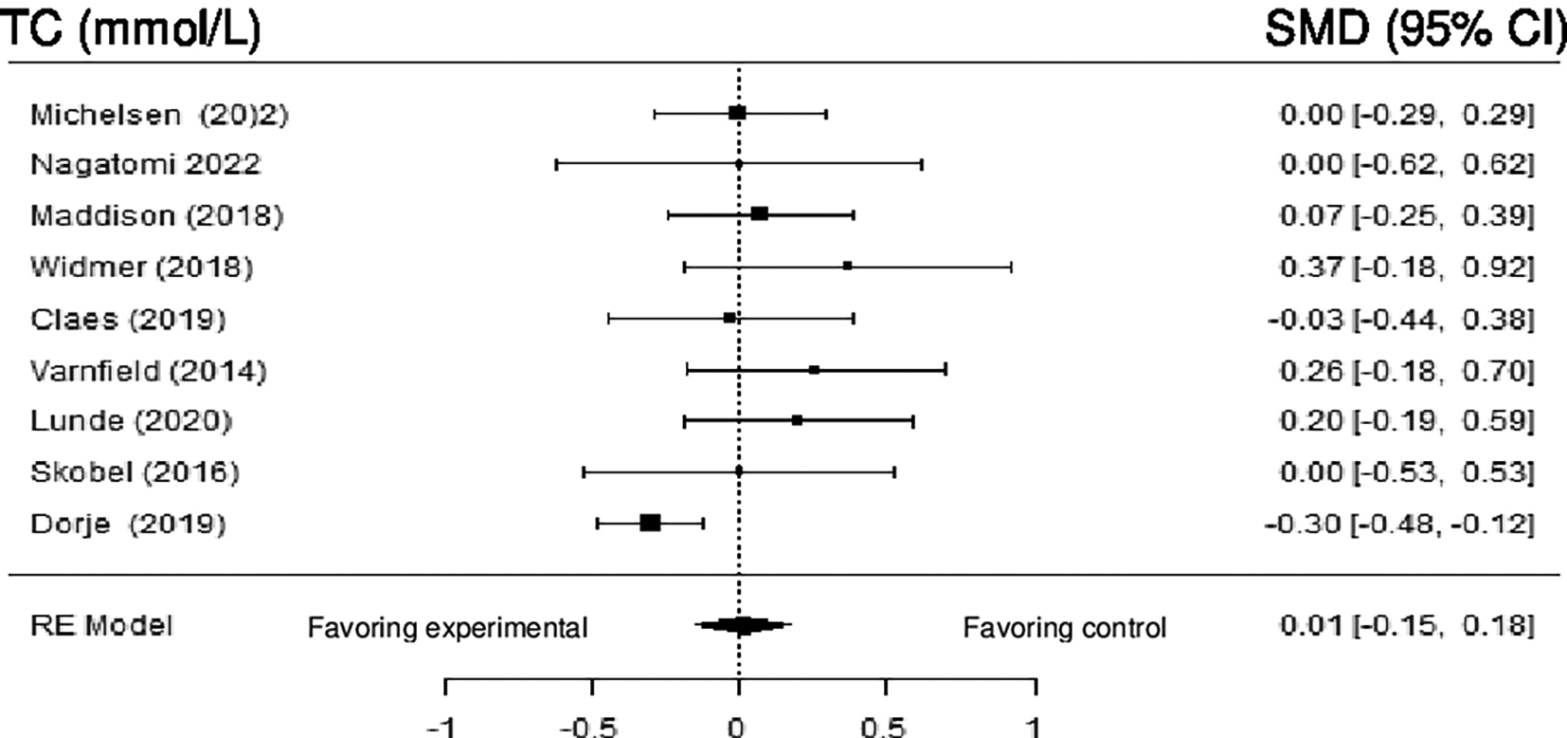
Forest plot of DHI vs CBCR/usual care impacts on TC.

**Total cholesterol:** Nine studies reported the impact of DHI on TC. Meta-analysis revealed no statistically significant difference (SMD = 0.09; 95% CI: -0.05 to 0.23) (z = 1.23, p = 0.22). Moderate heterogeneity was observed (Q (9) = 13.73, p = 0.13, tau ^2^ = 0.02, I ^2^ = 39.16%). Employing sensitivity analysis by removing the study by Dorje et al. (2019) made the heterogeneity null (Q (7) = 2.77, p = 0.9054, tau ^2^ = 0.0000, I ^2^ = 0.0000%), but without statistical significance. Figure 6 shows the forest plot of the impact of DHI vs CBCR/usual care on TC.

**Figure 7.**
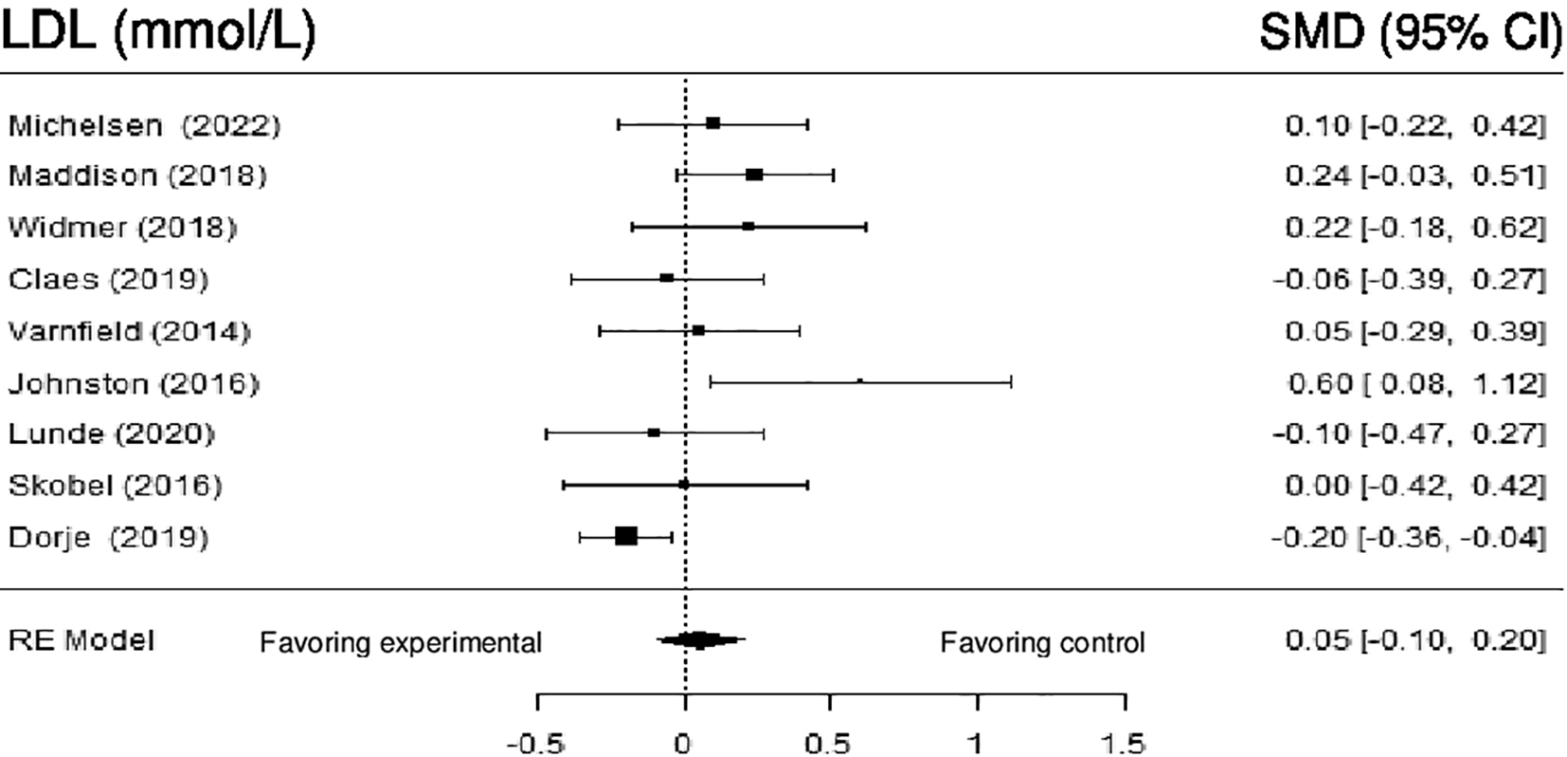
Forest plot of DHI vs CBCR/usual care impacts on LDL.

**Low density lipoprotein:** Nine studies reported the impact of DHI on LDL. Meta-analysis revealed no statistically significant difference (SMD = -0.01; 95% CI: -0.26 to 0.24) (z = -0.12, p = 0.91). A high amount of heterogeneity was noted among the results (Q (8) = 28.04, p = 0.0005, tau ^2^ = 0.10, I ^2^ = 71.09%). Employing sensitivity analysis by removing the studies by Dorje et al. (2019) and Johnston et al. (2016) made the heterogeneity null (Q (6) = 5.4898, p = 0.48, tau ^2^ = 0.0000, I ^2^ = 0.0000%), but without statistical significance (z = 1.61, p = 0.11). Figure 7 shows the forest plot of the impact of DHI vs CBCR/usual care on LDL.

**Figure 8.**
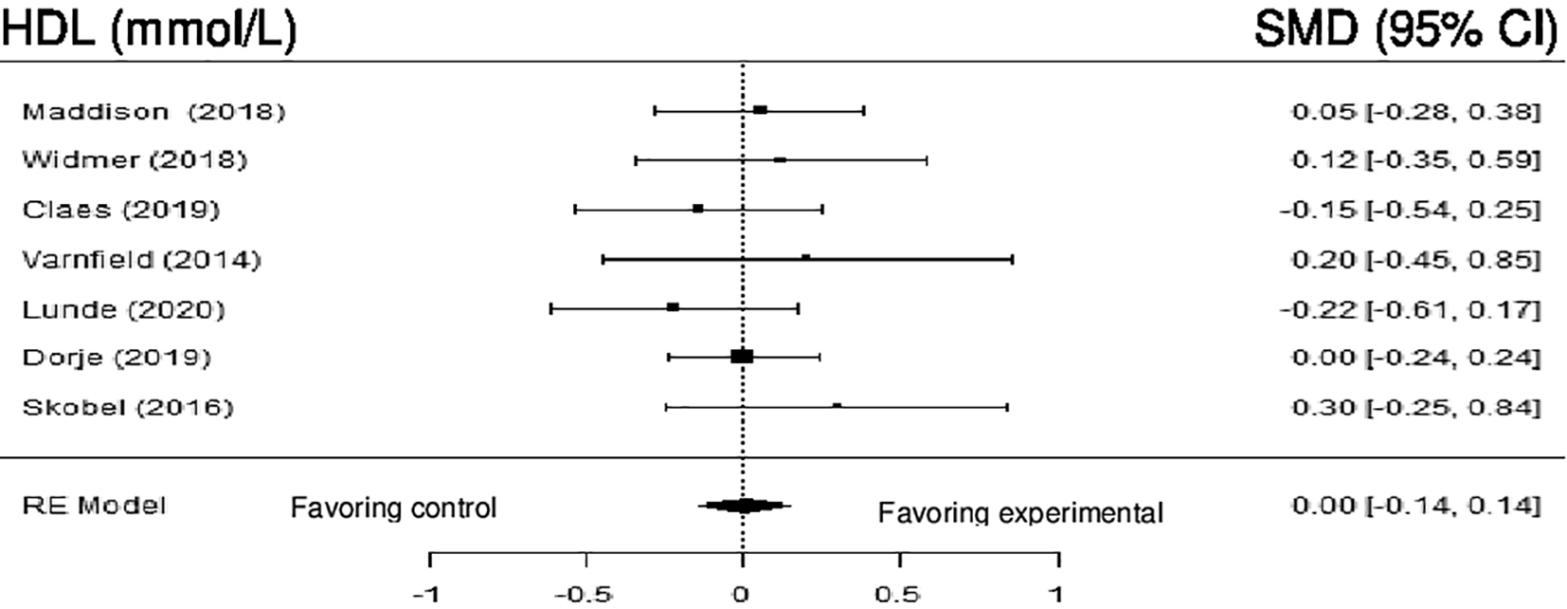
Forest plot of DHI vs CBCR/usual care impacts on HDL.

**High density lipoprotein:** Seven studies evaluated the impact of DHI on HDL. Meta-analysis showed no statistically significant difference (SMD = 0.01; 95% CI: -0.04 to 0.05) (z = 0.25, p = 0.81), and the heterogeneity was null (Q (6) = 3.68, p = 0.7205, tau ^2^ = 0.0000, I ^2^ = 0.0000%). Figure 8 shows the forest plot of the impact of DHI vs CBCR/usual care on HDL.

**Figure 9.**
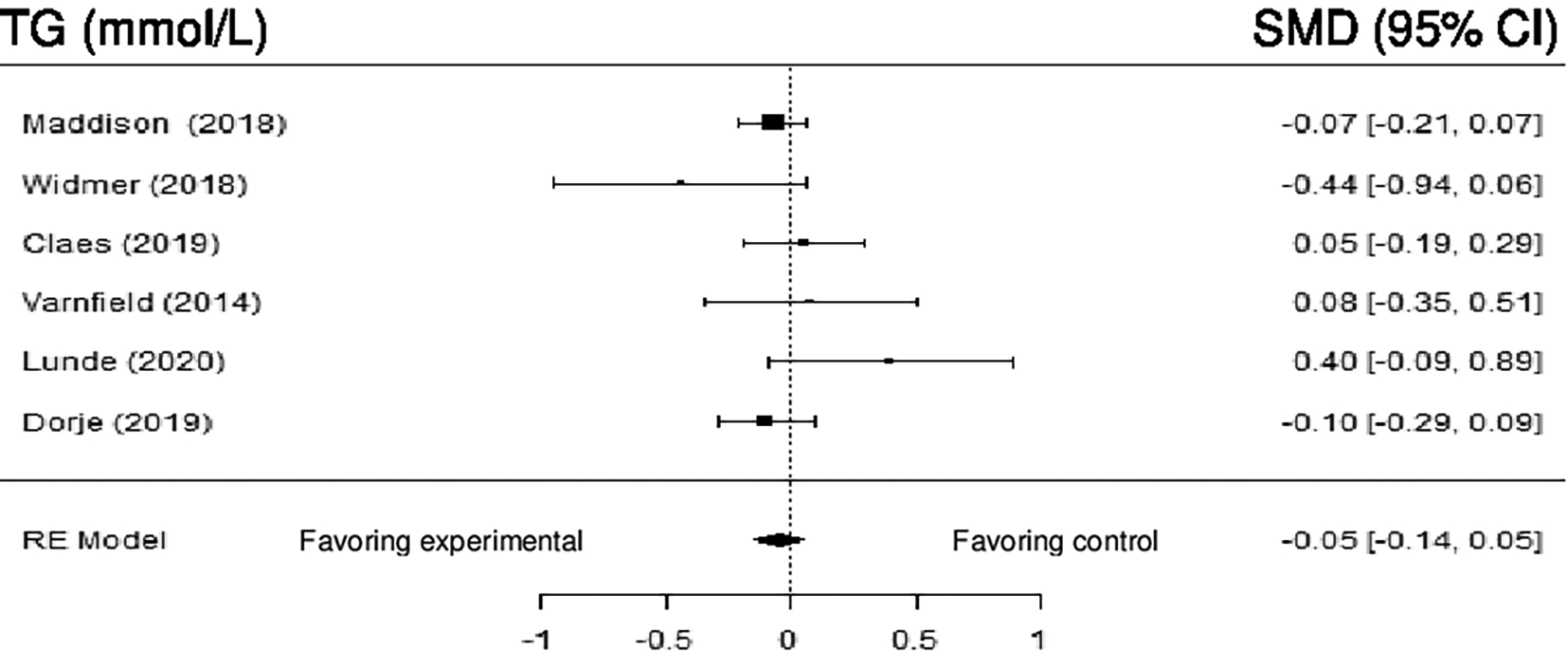
Forest plot of DHI vs CBCR/usual care impacts on TG.

**Triglyceride:** Six studies evaluated the impact of DHI on TG. Meta- analysis showed no statistically significant difference (SMD = -0.05; 95% CI: -0.14 to 0.05) (z = -0.95, p = 0.34). However, there was no significant heterogeneity Q (5) = 6.89, p = 0.23, tau ^2^ = 0.0000, I ^2^ = 0.00%). Figure 9 shows the forest plot of the impact of DHI vs CBCR/usual care on TG.

*Blood glucose*

**Figure 10.**
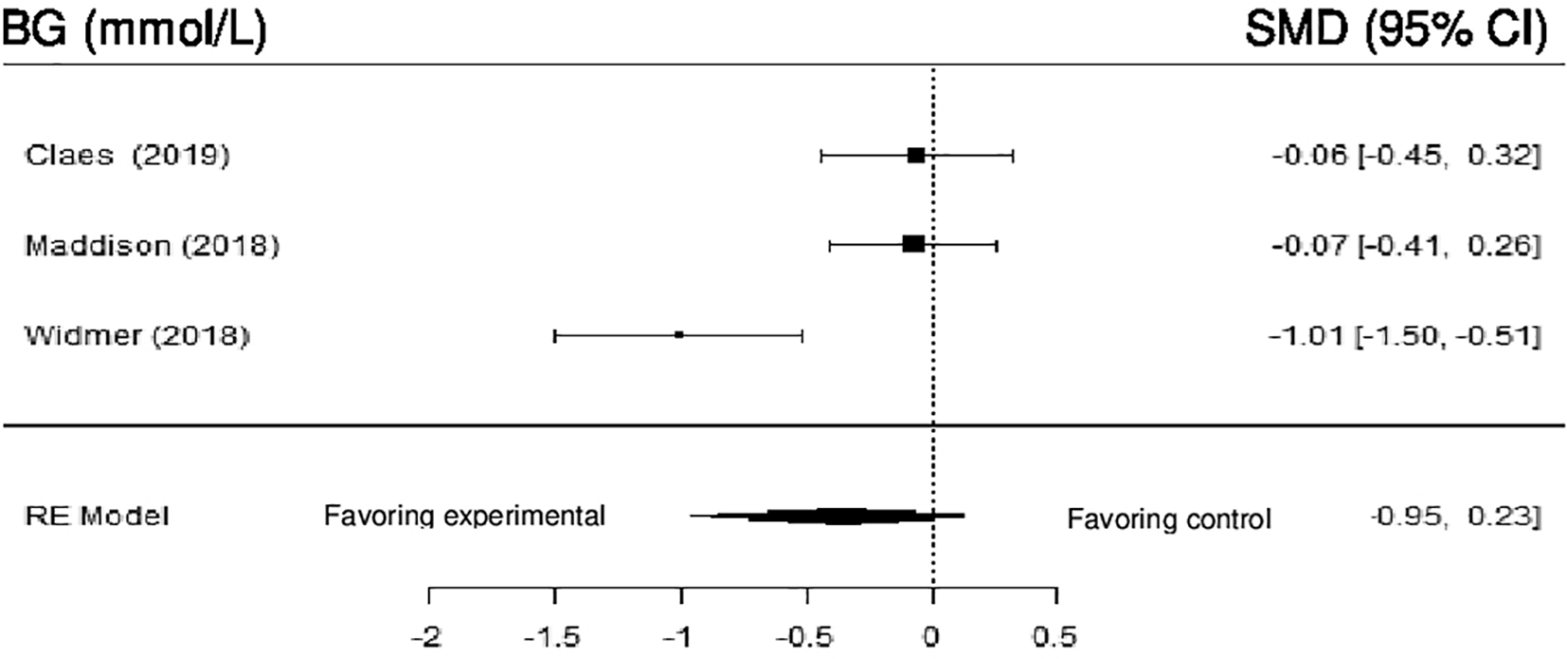
Forest plot of DHI vs CBCR/usual care impacts on BG.

Only three studies evaluated the impact of DHI on BG compared to control. Meta-analysis showed no statistically significant difference (SMD = -0.48; 95% CI: -1.14 to 0.19) (z = -1.41, p = 0.16), with high heterogeneity (Q (2) = 7.92, p = 0.0190, tau ^2^ = 0.2506, I ^2^ = 73.07%). Figure 10 shows the forest plot of the impact of DHI vs CBCR/usual care on BG.

*Physical capacity*

**Figure 11.**
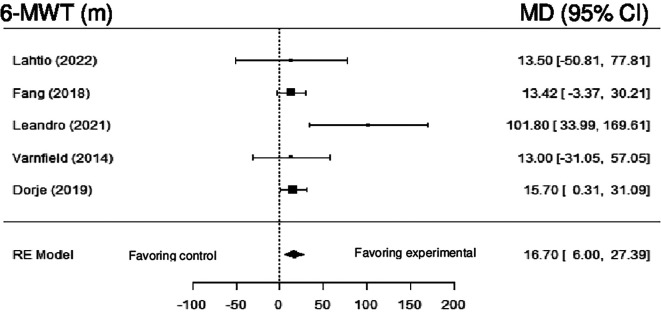
Forest plot of DHI vs CBCR/usual care impacts on 6-MWT.

**Six-Minute Walk Test (6-MWT):** Five studies examined the effect of DHI on 6-MWT. Meta-analysis showed statistically significant differences (MD = 16.70; 95% CI: 6.00 to 27.39) (z = 3.06, p = 0.00), with null heterogeneity (Q (4) = 6.25, p = 0.18, tau ^2^ = 0.0000, I ^2^ = 0.0000%). Figure 11 shows the forest plot of the impact of DHI vs CBCR/usual care on 6-MWT.

**Figure 12.**
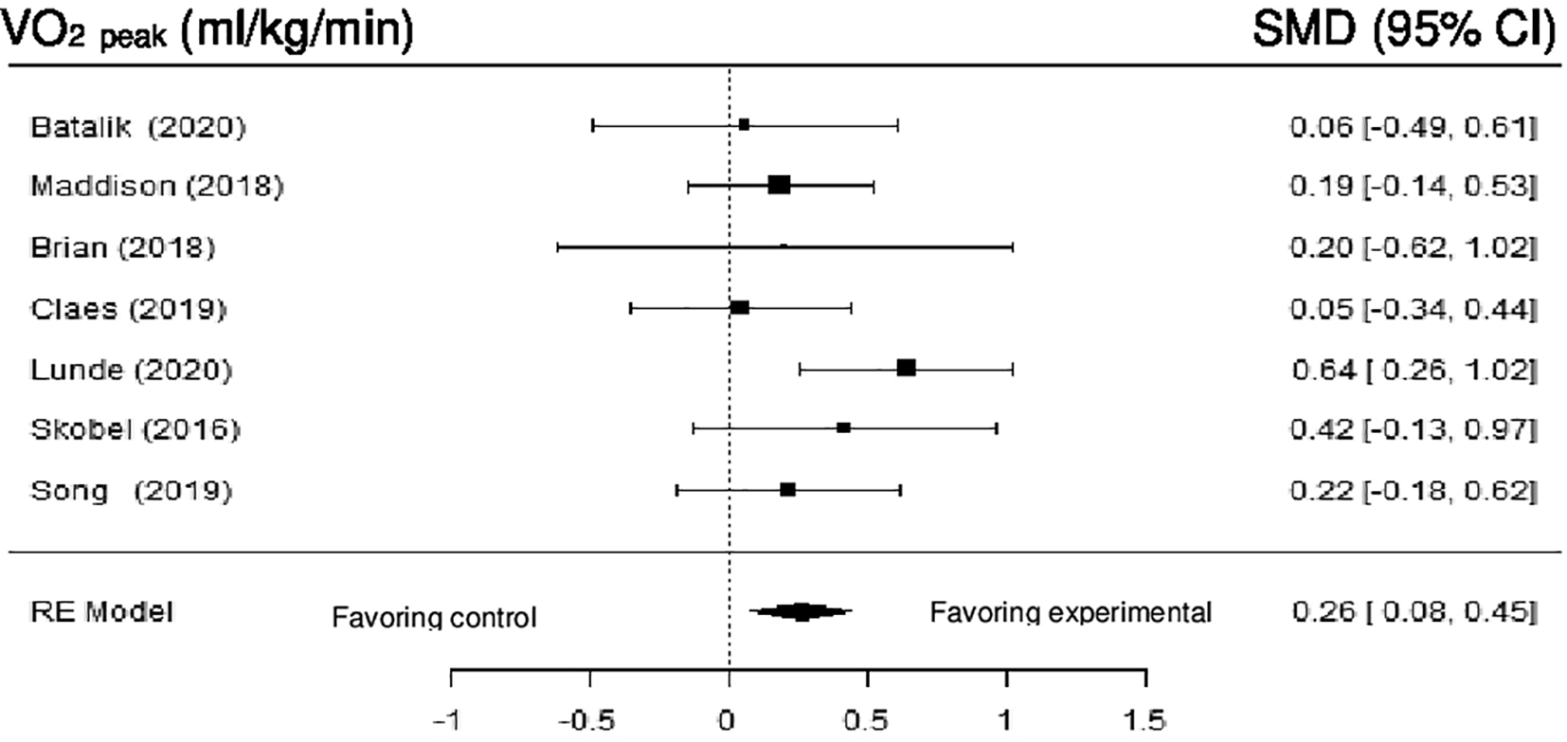
Forest plot of DHI vs CBCR/usual care impacts on VO2 peak.

**Maximum oxygen consumption:** Seven studies examined the impact of DHI on VO _2 peak_. Meta-analysis showed statistically significant differences (SMD = 0.27; 95% CI: 0.08 to 0.45) (z = 2.85, p = 0.0044), with low heterogeneity in the true outcomes (Q (6) = 6.02, p = 0.42, tau ^2^ = 0.01, I ^2^ = 15.27%). Figure 12 shows the forest plot of the impact of DHI vs CBCR/usual care on VO _2 peak_.

*Body mass index*

**Figure 13.**
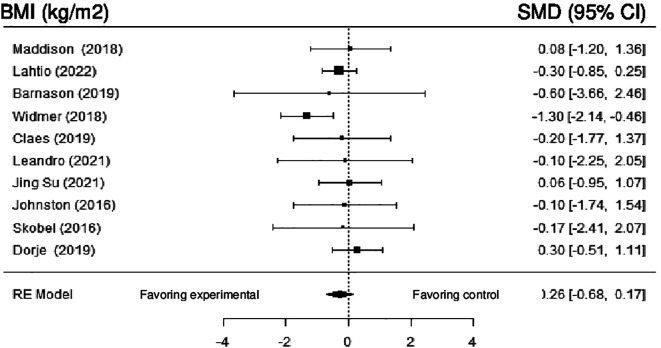
Forest plot of DHI vs CBCR/usual care impacts on BMI.

Ten studies evaluated the impact of DHI on BMI. Despite the non-significant difference (MD = -0.05; 95% CI: -0.17 to 0.07) (z = -0.77, p = 0.44), the results showed a trend toward experimental groups with neglected heterogeneity (Q (9) = 9.77, p = 0.37, tau ^2^ = 0.0001, I ^2^ = 0.34%). Figure 13 shows the forest plot of the impact of DHI vs CBCR/usual care on BMI.

*Quality of life*

**Figure 14.**
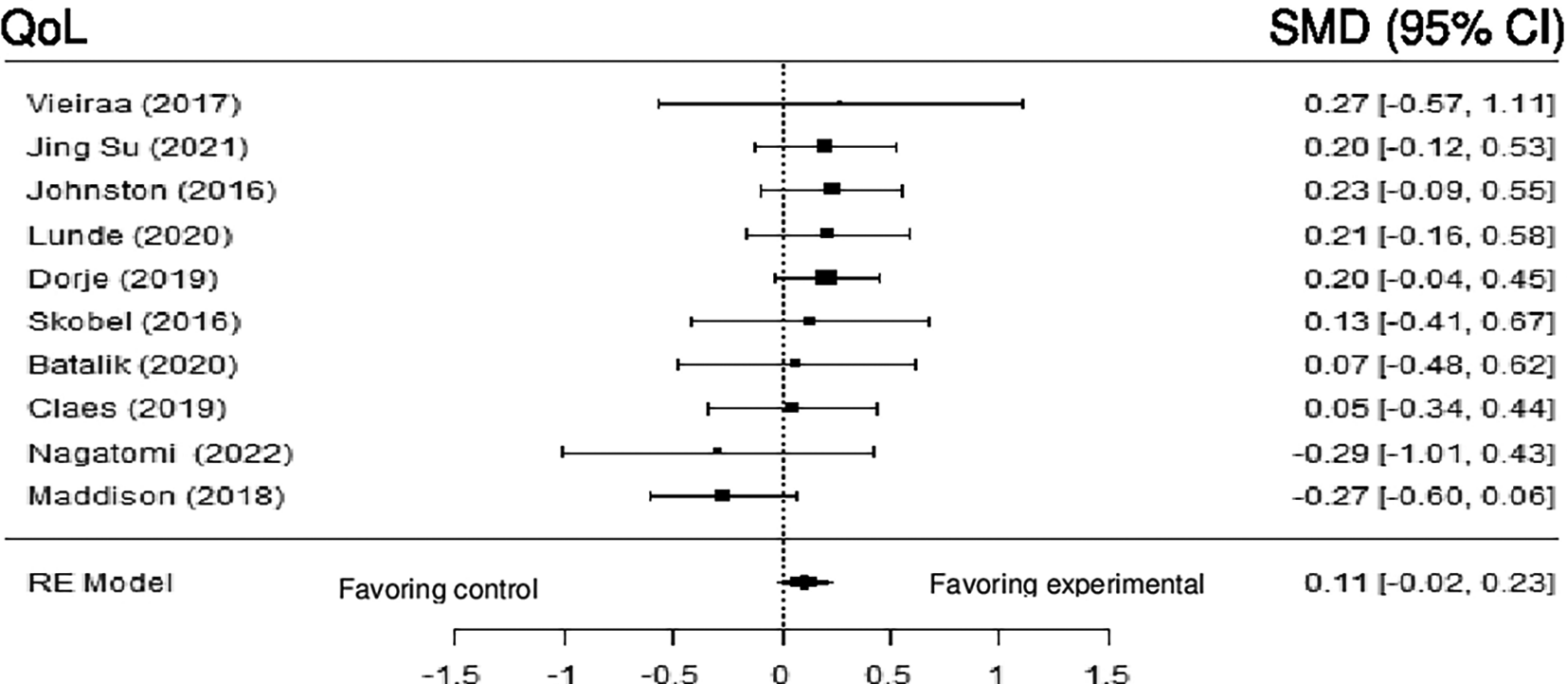
Forest plot of DHI vs CBCR/usual care impacts on QoL.

**Figure 15.**
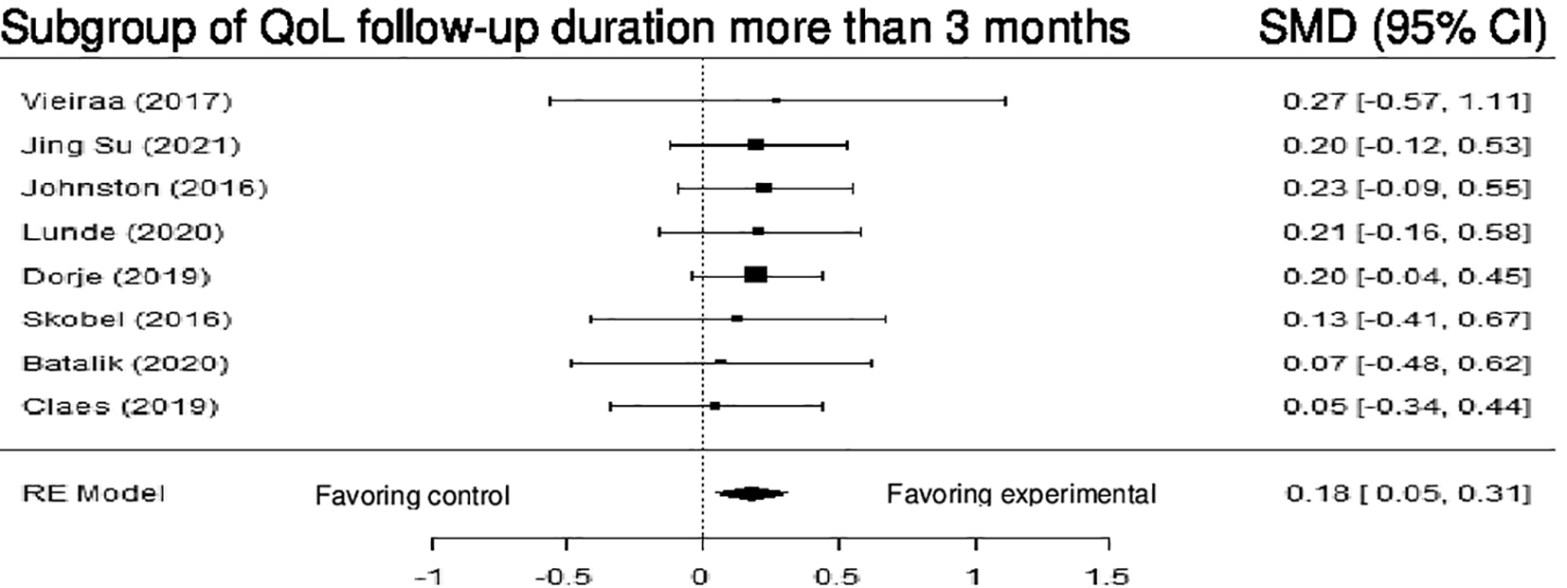
Forest plot of subgroup analysis of DHI vs CBCR/usual care impacts on QoL (follow-up duration >3 months).

**Figure 16.**
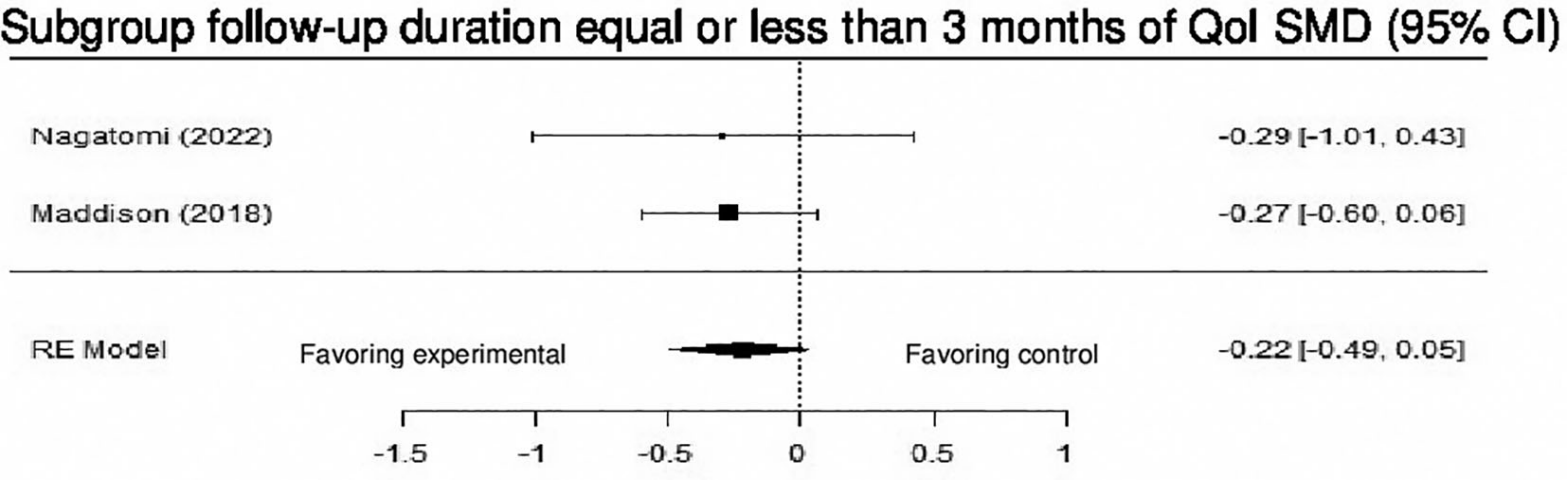
Forest plot of the subgroup analysis of DHI vs CBCR/usual care impacts on QoL (follow-up duration ≤ 3 months).

Ten studies examined the impact of DHI on QoL versus control. Although the results revealed no statistically significant difference (SMD = 0.10; 95% CI: -0.02 to 0.23) (z = 1.62, p = 0.11), a trend favoring intervention was observed. Subgroup analysis based on duration of follow-up (≤ 3 months vs > 3 months) was conducted, and a statistically significant difference was observed in the subgroup with duration of follow-up >3months (SMD = 0.18; 95% CI: 0.05 to 0.31) (z = 2.71, p = 0.00), and the heterogeneity was null (Q (7) = 0.84, p = 0.9970, tau ^2^ = 0.0000, I ^2^ = 0.0000%). while the studies with duration of follow-up ≤ 3 months did not demonstrate any significant difference. Figure 14 shows the forest plot of the impact of DHI vs CBCR/usual care on QoL. ( Figure 15) and ( Figure 16) show the forest plots of the subgroup analysis of the impact of DHI vs CBCR/usual care on QoL

## Discussion

Individuals living with CVD face constant vulnerability to adverse events. The presence of uncontrolled risk factors significantly heightens the likelihood of experiencing major adverse cardiac and cerebrovascular events (MACCE). To address preventable factors (e.g., overweight, hyperlipidemia, hyperglycemia, and hypertension), CR has emerged as a well-established traditional intervention. However, despite its documented effectiveness in reducing mortality and readmission rates, conventional center-based CR (CBCR) experiences challenges in terms of adherence. Studies reveal a concerning statistic, with less than 20% of patients actively participating in CBCR. Various reasons have been cited for this diminished participation, including logistical factors such as distance and transportation, as well as motivational issues. ^8^^,^^9^

Recognizing these barriers, DHI has emerged as an innovative method to overcome these challenges. By leveraging digital technologies, this approach aims to enhance accessibility, convenience, and motivation, thereby offering a promising solution to improve adherence rates and optimize the overall impact of CR for individuals with CVD.

Maximal oxygen consumption is a pivotal physiological metric gauging an individual’s upper limit for oxygen utilization during intense physical exertion. ^46^ VO _2 peak_ serves as a vital indicator of cardiovascular endurance and fitness. ^47^^,^^48^ This parameter holds noteworthy importance in exercise physiology, managing exercise prescription, and weighing the efficiency of intervention programs. ^49^ The strong opposite relationship between VO _2 peak_ and CVD mortality has been documented in many large-scale studies. ^50^

In a large prospective study conducted in the UK, physical capacity emerged as a robust predictor of mortality, surpassing traditional cardiac risk factors. ^51^ Consequently, the findings of the current review confirm these contemporary conclusions, stressing the need for intervention programs aimed at increasing physical capacity, and providing valuable insights into strategies that can positively enhance overall health.

In conjunction with VO _2 peak_, the 6-MWT offers a practical and insightful assessment of functional capacity and endurance, tasking individuals with covering as much distance as possible in a six-minute timeframe. ^52^ Notably, the effect estimate in all included studies evaluated the impact of DHI on VO _2 peak_ and 6-MWT outcomes favored the experimental groups. Despite the variation in the nature and duration of the interventions, it has been observed that the heterogeneity among studies evaluating VO _2 peak_ was minimal, and null among the studies that evaluated 6-MWT. This finding underscores the efficacy and consistency of DHI in enhancing cardiovascular fitness, showcasing their potential to surpass conventional modalities.

The superiority of DHIs over conventional programs may be interpreted by the frequent reminder and feedback features available in the former. These features serve as warning signs for the patient to increase adherence to the program, and devices such as pedometers, ECG, and HR monitoring directly transfer patients’ data to the healthcare provider, whereby they can instantly (or rapidly) intervene, encourage, and provide required instructions. Similarly, a recent literature review revealed that a statistically significant improvement in physical capacity was observed when using DHI compared with traditional care approaches. ^53^ This finding contradicts the result of another meta-analysis which did not find a significant impact on VO _2 peak_, the forest plot in that study indicated improvement in intervention groups, but the result may be due to the small number of included studies (n = 4). ^54^

While the majority (7/10) of the studies investigating the influence of DHI on BMI demonstrated favor toward the experimental groups, the pooled effect did not yield statistically significant differences. Similarly, non-significant effects of DHI on BMI have been observed in previous studies. ^54^^,^^55^ Nevertheless, the comparable effects observed between DHI and CBCR or usual care on BMI with very low heterogeneity (I ^2^ = 0.34%) could be attributed to the influence of other factors not adequately addressed in both types of interventions, such as metabolic rate and sleeping pattern. ^56^ Therefore, it is crucial to consider these multifaceted influences when evaluating the effectiveness of DHI in the context of BMI outcomes.

The meta-analysis results indicate a non-significant difference between DHI and CBCR or usual care in TC, LDL, HDL, and TG levels. Various factors, such as diverse modalities within DHI, patient adherence, uptake, duration and intensity of interventions, average age, and percentages of males and females in each study, may be instrumental in the lack of statistically significant results. Moreover, the limited positive effects on lipid profile may be because DHIs might not effectively address symptoms and medication management. ^57^ Improving these aspects in the intervention could lead to better physiological outcomes. ^37^

At initial analysis, this review showed no significant differences between the two types of intervention on SBP and DBP, marked by high heterogeneity among the included studies. After conducting the sensitivity analysis by removing two studies (Dorje et al., 2019; Skobel et al., 2017) from SBP outcomes, and one study from diastolic outcomes (Skobel et al., 2017), the results became statistically significant, with moderate heterogeneity, favoring the experimental group.

Hypertension is a known cardiometabolic risk factor associated with increased morbidity and mortality. ^58^^–^^61^ According to a study by Pan, H., et al (2020) there was a two-fold increase in risk for sudden cardiac death with prevalent hypertension, and a 28% increase in risk for sudden cardiac death per 20 mmHg increment in SBP. ^62^ The comprehensive nature of DHI extends beyond exercise routines, encompassing dietary guidance, stress management, and adherence to a healthy lifestyle. This holistic approach addresses various contributors to blood pressure control, promoting a more effective and tailored intervention. Additionally, direct monitoring and instant feedback enhance the patients’ adherence and enable early professionals’ intervention. However, controversial results have been demonstrated in previous studies, for instance, one study observed a significant reduction in blood pressure for those who received DHI compared to those who did not, ^63^ and another study demonstrated no statistically significant differences for these variables. ^54^

High blood glucose level is a risk factor leading to CVD, and is associated with all-cause mortality. ^64^ Only three studies evaluated the impact of DHI on glucose level, and the meta-analysis revealed no significant differences, with high heterogeneity. However, the three studies favored the experimental groups. Similarly, no significant difference was observed in a previous meta-analysis of three studies. ^65^ The scarce number of studies examining the impact of DHI on blood glucose warrants more future investigation.

The pooled intervention effect on QoL showed no significant differences, with low heterogeneity. Subgroup analysis based on the duration of follow-up (> 3 months vs ≤ 3 months) yielded a statistically significant pooled effect favoring the experimental groups in studies with follow-up duration > 3 months, with null heterogeneity, while the studies with duration of follow-up ≤ 3 months did not demonstrate any significant difference. This result indicated that DHIs were more effective with longer periods. Notably, there was consistent agreement among these studies, as evidenced by the absence of heterogeneity, and the finding that patient adherence and engagement with a new intervention is a gradual process, often taking some time. ^66^ Additionally, the integration of DHI necessitates adaptation and technology familiarity that may take more time, particularly with patients with advanced age. ^67^ Inconsistent findings have been observed in the previous studies, one of which found a positive impact of using DHI on QoL, ^68^ while another did not observe superiority of DHI on QoL compared to CBCR or usual care. ^54^ However, a small number of included studies were analyzed in both of these previous reviews.

These results strongly suggest that DHI is a potential feasible solution for certain rehabilitation program participants who experience certain difficulties of access. For instance, in conventional formats are not accessible or realistic for such participants, DHI can achieve better outcomes in terms of physical activity, blood pressure and QoL on the long term. Additionally, similar outcomes can be achieved in terms of BMI and lipid profile indicators, highlighting the potential effectiveness of an efficient potential healthcare service augmented by modern technologies, to optimize the achievement of clinical targets. This is in alignment with the emerging healthcare service paradigmatic context, seeking to maximize patient satisfaction and outcomes in particular (and important) areas such as CVD.

Some limitations have been encountered in this review. Firstly, the small number of participants in some included studies limited the statistical power and increased the amount of heterogeneity in particular outcomes. Secondly, the DHIs were vary in terms of intensity and duration so the results should be interpreted with caution in this context. Thirdly, the generalizability of the result may be affected by the higher proportional representation of males than females in included studies. However, this may refer to CVD prevalence rather than study design.

## Conclusion

The findings emphasize the significant impact of DHI vs CBCR or usual care methods on physical capacity, as evidenced by improvement in VO _2 peak_ and the 6-MWT. Significant differences have been observed favored DHI in systolic and diastolic BP employing sensitivity analysis. Despite the non-statistically significant differences in BMI and lipid profile, the comparable effect between the two methods suggests the superiority of DHI over CBCR or usual care due to its convenient nature, accessibility, and cost-effectiveness. The positive effects on QoL, especially with extended engagement, highlight the gradual yet meaningful impact of DHI compared with CBCR or usual care methods. No statistically significant difference was observed in relation to the impact on blood glucose.

## Data Availability

No data associated with this article. The Endnote reference manager available from: https://endnote.com/downloads/ alternative free access reference manager available from: https://www.mendeley.com/download-reference-manager/windows. Meta-analysis statistical spreadsheet available from: https://www.jamovi.org/download.html. Risk of bias assessment available from: https://sites.google.com/site/riskofbiastool/welcome/rob-2-0-tool/current-version-of-rob-2?authuser=0. Zenodo: Data set for article “Digital health intervention in patients undergoing cardiac rehabilitation: systematic review and meta-analysis”, https://doi.org/10.5281/zenodo.11360997. ^69^ Data are available under the terms of the Creative Commons Zero “No rights reserved” data waiver (CC0 1.0 Public domain dedication). Zenodo: PRISMA Checklist for Article “ Digital Health Intervention in Patients Undergoing Cardiac Rehabilitation: Systematic Review and Meta-Analysis. https://doi.org/10.5281/zenodo.11288634 . ^70^ Data are available under the terms of the Creative Commons Zero “No rights reserved” data waiver (CC0 1.0 Public domain dedication). Zenodo: PRISMA flow chart for Article “ Digital Health Intervention in Patients Undergoing Cardiac Rehabilitation: Systematic Review and Meta-Analysis”. https://doi.org/10.5281/zenodo.11288735 . ^71^ Data are available under the terms of the Creative Commons Zero “No rights reserved” data waiver (CC0 1.0 Public domain dedication).
