# Supplementary material for: Digital health intervention in patients undergoing cardiac rehabilitation: systematic review and meta-analysis
Source: F1000Res. 2024 Jun 7;13:596. [Version 1] doi: 10.12688/f1000research.152315.1 (PMC11231633; doi:10.12688/f1000research.152315.1)
Supplement: Peer review history

# Reviewer report: Reviewer response for version 1

**DOI:** 10.5256/f1000research.167060.r292594

**Published:** 2024-07-08

**Authors:** Issa Hweidi

**Affiliations:** 1 Jordan University of Science and Technology, Irbid, Irbid Governorate, Jordan

Very-well written paper that needs some additional improvements to get it ready for indexing. The following are my general comments to the authors: 

**ABSTRACT**

 -A structured summary has been written.

 -Done between 2000 and 2023.

 -Keywords should be checked according to MESH guidelines.

**INTRODUCTION**

 -There seems to be a connection problem between Paragraph 1 and Paragraph 2.

 -References could be more up-to-date. There are current sources where the same statements are written. It also needs to be revised grammatically.

**METHODS**

 -Inclusion and exclusion criteria determined according to PICO stages

 -In the -Study selection section, it is necessary to indicate who selected the studies. Was there any bias or not? The reader should get clear answers to these questions in this section.

 -There are semantic problems in English up to this point. English language editing is required.

 -Meta-analysis part done very well.

 -Please describe the results of the critical appraisal of the quality of the selected studies.

 -Please provide more detail regarding the intervention protocols and their effectiveness

**DISCUSSION**

 - What is expressed in the method section should not be rewritten in this section.

 - p values should be written in a standardized way.

 - More recent references should be used.

 - Please discuss the clinical implications of the study results in clinical practice.

Are the rationale for, and objectives of, the Systematic Review clearly stated?

Yes

Is the statistical analysis and its interpretation appropriate?

Yes

If this is a Living Systematic Review, is the ‘living’ method appropriate and is the search schedule clearly defined and justified? (‘Living Systematic Review’ or a variation of this term should be included in the title.)

Not applicable

Are sufficient details of the methods and analysis provided to allow replication by others?

Partly

Are the conclusions drawn adequately supported by the results presented in the review?

Yes

Reviewer Expertise:

Cardiac Nutsing

I confirm that I have read this submission and believe that I have an appropriate level of expertise to confirm that it is of an acceptable scientific standard.

# Reviewer report: Reviewer response for version 1

**DOI:** 10.5256/f1000research.167060.r292601

**Published:** 2024-06-26

**Authors:** Abedalmajeed Methqal Shajrawi

**Affiliations:** 1 Faculty of Nursing, Higher Colleges of Technologies,, Abu Dhabi, United Arab Emirates

The manuscript is well written, organized and well structured. 

 The abstract well written, clear and informative. 

 Introduction: Write more about why cardiac patients do not engage in CR. what are the other solutions. 

 give a brief about digital health. benefits and disadvantages. etc.

 Method: Clear and well-written. 

 Results: Perfect, this section is concise and informative.

 Discussion: you need to mention which stage in CR that used in each study. Also, the difference in cultural impacts on the health lifestyle . 

 Conclusion: need to be revised.

Are the rationale for, and objectives of, the Systematic Review clearly stated?

Yes

Is the statistical analysis and its interpretation appropriate?

Yes

If this is a Living Systematic Review, is the ‘living’ method appropriate and is the search schedule clearly defined and justified? (‘Living Systematic Review’ or a variation of this term should be included in the title.)

No

Are sufficient details of the methods and analysis provided to allow replication by others?

Yes

Are the conclusions drawn adequately supported by the results presented in the review?

Yes

Reviewer Expertise:

my interest areas are Cardiovascular disease, risk factors, psychological variables  and physical activity.

I confirm that I have read this submission and believe that I have an appropriate level of expertise to confirm that it is of an acceptable scientific standard.
